# Beyond the Hype: (How) Are Work Regimes Associated with Job Burnout?

**DOI:** 10.3390/ijerph20043331

**Published:** 2023-02-14

**Authors:** Kristen du Bois, Philippe Sterkens, Louis Lippens, Stijn Baert, Eva Derous

**Affiliations:** 1 Faculty of Economics and Business Administration, Ghent University, 9000 Ghent, Belgium; 2 Faculty of Psychological and Educational Sciences, Ghent University, 9000 Ghent, Belgium; 3 Faculty of Social Sciences, Vrije Universiteit Brussel, 1050 Brussels, Belgium; 4 Research Foundation Flanders, University of Antwerp, 2000 Antwerp, Belgium; 5 Institute of Labor Economics (IZA), Université Catholique de Louvain, 1348 Ottignies-Louvain-la-Neuve, Belgium

**Keywords:** burnout, work environment, Burnout Assessment Tool, job demands–resources theory, burnout prevention, work regime, part-time work

## Abstract

Job burnout affects countless workers and constitutes a major issue in working life. Prevention strategies such as offering part-time options and shorter working weeks have been widely advocated to address this issue. However, the relationship between shorter work regimes and burnout risk has not yet been investigated across diverse working populations applying validated measures and frameworks for job burnout. Building on the most recent operationalisation of job burnout and the seminal job demands–resources theory, the purpose of the current study is to investigate whether shorter work regimes are associated with lower burnout risk and whether the job demands–resources explain this association. To this end, a heterogenous sample of 1006 employees representative for age and gender completed the Burnout Assessment Tool (BAT) and Workplace Stressors Assessment Questionnaire (WSAQ). Our mediation analyses yield a very small but significant indirect association between work regimes and burnout risk through job demands, but no significant total or direct association between work regimes and burnout risk. Our result suggests that employees in shorter work regimes experience slightly fewer job demands, but are equally prone to developing burnout as their full-time counterparts. The latter finding raises concerns about the sustainability of burnout prevention that focuses on mere work regimes instead of the root causes of burnout.

## 1. Introduction

In the recent review study on the state of burnout research, Demerouti et al. state that job burnout affects millions of individuals and represents a fundamental challenge in working life [1]. Despite the wide variety of available burnout conceptualisations [1], the World Health Organisation has recently recognised burnout as “an occupational phenomenon resulting from chronic workplace stress that has not been successfully managed” [2]. The core symptom that employees at (high) risk of burnout typically experience is extreme exhaustion [1].

Schaufeli roughly estimates that the prevalence of burnout was 10% for the European workforce in 2015 [3]. Recent international post-lockdown studies find similar results across measurement methods. For example, a recent post-lockdown study calculates an overall burnout risk of 11% [4] using the Burnout Assessment Tool [5]. Since burnout has been primarily associated with sickness absence, occupational injuries and accidents, poor work performance and reduced productivity [6], prevention remains a burning issue for workers, employers and society at large.

### 1.1. Job Demands-Resources

Demerouti et al. state that effective prevention should focus on redesigning the workplace and removing the causes of burnout [1]. According to the seminal work of Bakker and Demerouti [7] on this matter, two independent processes can lead to the development of burnout. On the one hand, burnout may result from a health impairment process that departs from high job demands, i.e., aspects of work that require employee effort and may result in physical or psychological costs. Common examples of job demands are work and time pressure [1,7]. On the other hand, burnout may result from a declined motivational process that departs from reduced job resources, which are the aspects of work that are energising, facilitate goal achievement, or enable personal development. Common examples of job resources are support, appreciation and autonomy [1,7]. In line with these core causes of burnout, customary organisational interventions for burnout focus on reducing work stressors and enhancing organisational resources available to employees [8].

### 1.2. Shorter Work Regimes

One specific intervention in this context is the modification of exposure time to potential stressors, e.g., by reducing the time the worker is exposed to stressful elements [8]. Relatedly, offering part-time options and shorter working weeks to employees are increasingly advocated as strategies to prevent burnout [8,9,10,11,12].

Remarkably, a systematic search of peer-reviewed intervention studies on this matter provides no conclusive evidence for negative effects of shorter work regimes on burnout. More specifically, only one small sample study (*n* = 28) among Swedish social workers finds lower levels of emotional exhaustion after a 25% working time reduction [13]. Conversely, an adjacent longitudinal intervention study on limiting working hours [14] suggest that working time reduction may not per se reduce the risk of burnout [15]. However, at present, the relationship between work regimes and burnout remains relatively unexplored territory.

### 1.3. Work Regimes and Burnout

In line with the latter, current literature provides no conclusive evidence that shorter work regimes are generally associated with lower burnout risk. On the one hand, one study among 345 full-time and 77 part-time physicians with a single-item measure of burnout concluded that part-time workers report less burnout [9]. On the other hand, a recent study with more extensive burnout measures (Copenhagen Burnout Inventory) found no objective differences in burnout risk among 408 full-time and 190 part-time physicians [15]. Similar to the latter findings, a recent study among 6109 full-time and 5905 part-time teachers found that part-time and full-time teachers do not differ in their risk of burnout (measured via Maslach Burnout Inventory) [16]. To our knowledge, however, there are no recent studies investigating the relationship between work regimes and burnout outside these specific job domains. Since (i) the well-being effects of these working conditions may vary between jobs [17] and (ii) previous studies on work regimes and burnout provide mixed findings, the relationship between work regimes and burnout needs further investigation in more heterogeneous working populations.

Taken together, this study’s general aim is to further explore the relationship between work regimes and burnout and investigate whether shorter work regimes are associated with lower burnout risk in a more heterogenous sample of workers. Therefore, our first research hypothesis reads as follows.

**Hypothesis 1.**  *There is a significant association between work regimes and burnout risk in general.*

Following the recommendations to apply nuanced theoretical frameworks to explain relationships between aspects of working time and well-being [17], our study also aims to identify possible explanatory factors in the association between work regimes and burnout. Therefore, our study adds to the existing literature in two additional ways. First, to our knowledge, our study is the first to build on the most novel validated burnout conceptualisation [5] to investigate the association between work regimes and burnout. This conceptualisation takes into account all four core symptoms that employees at risk of burnout may experience: exhaustion, mental distance, emotional impairment and cognitive impairment [5]. The accompanying measurement instrument (Burnout Assessment Tool) measures total burnout risk in a nuanced matter, including factors for all four core symptoms [5]. Second, as elaborated in section “1.1. Job demands—resources”, job demands and resources play a crucial role in the development of burnout. Even more so, previous research suggested that job demands (e.g., time pressure) and resources (e.g., poor support from colleagues and leaders) may mediate the association between (marginal) part-time work and health [18]. Therefore, our study considers the job demands–resources theory on the causes of burnout [1,7] as a mediating mechanism to elucidate potential associations between work regimes and burnout risk. We additionally include the following two hypotheses.

**Hypothesis 2.**  *There is a significant indirect association between work regime and burnout risk among employees through job demands.*

**Hypothesis 3.**  *There is a significant indirect association between work regime and burnout risk among employees through job resources.*

**Figure 1:**
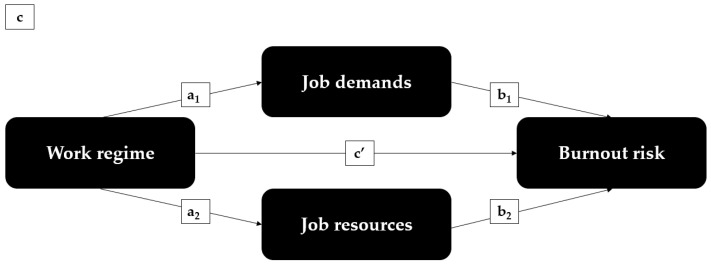
Conceptual framework.

Our three hypotheses converge into the conceptual framework presented in Figure 1. To test the first hypothesis, we estimate the total association (path c) between work regime and burnout and the residual direct association (path c’) after controlling for the mediators’ job demands and resources. To test the second and third hypothesis, the indirect associations through job demands (a_1_b_1_) and job resources (a_2_b_2_) are explored.

## 2. Materials and Methods

### 2.1. Sample Specification

We gathered a sample of 1006 Belgian employees across work regimes through a specialised research agency in June 2022. The sample was representative for both gender and age. More specifically, the sample comprised 493 (49%) men and 513 (51%) women with a mean age of 46.018 (SD = 12.305) years old. We pursued representativeness for these two sociodemographic variables because adjacent literature on the relationship between working time and well-being underlines their importance in this domain [17].

As displayed in Table 1, 714 participants (71%) worked full-time, and 292 participants (29%) worked part-time. Among the 292 part-time employees, 150 (51%) worked less than 80% of a full-time regime, and 142 (49%) worked a regime of 80% or more. The sample was heterogeneous in terms of sector, organisation size and job domain. More specifically, 709 (70%) participants worked in the private sector, whereas 297 (30%) worked in the public sector. Further, 503 (50%) participants worked in a small or medium organisation (with 250 employees or less) and 503 (50%) participants worked in a large organisation (with more than 250 employees). Table 1 displays the wide variety of job domains (22) in which the participants worked.

All participants completed an online questionnaire through the platform Qualtrics XM in June 2022. All participants were informed about the study’s purpose, as well as the anonymised processing and protection of their data administered through the platform Qualtrics XM. Informed consent was obtained in digital, written form prior to the start of the survey. This non-interventional survey study adhered to the General Ethical Protocol of the two faculties where the research took place (Faculty of Economics and Business Administration and the Faculty of Psychological and Educational Sciences at Ghent University). Therefore, ex ante ethical approval of this survey research, which was based upon prior and informed consent, was not obligatory.

### 2.2. Dependent Variable and Mediators

We used validated instruments to measure the outcome variable and mediators of our conceptual model. Although 88% of publications on burnout use the classic Maslach Burnout Inventory [5] to measure burnout, we chose to operationalise our primary outcome variable according to the most recent validated burnout conceptualisation of the Burnout Assessment Tool [5].

The choice for this instrument was motivated by three main reasons. First, the more recently developed Burnout Assessment Tool addresses the need for innovations related to (i) the conceptualisation of burnout and (ii) the psychometric and technical features of the Maslach Burnout Inventory, while simultaneously guaranteeing convergent validity with the latter (indicating that a similar concept is being measured) [5,19,20].

Second, this tool shows discriminant validity with other well-being constructs, such as work engagement and workaholism [5]. Third, the authors of the Burnout Assessment Tool have developed a shorter 12-item version (BAT-12) that was shown to have robust psychometric properties and can be used in an effective, valid way to measure employees’ burnout levels [20]. More specifically, the BAT-12 enables us to distill factor scores for all four core symptoms of burnout (exhaustion, mental distance, emotional impairment and cognitive impairment) as well as a total risk score that can be used as an overall indicator for job burnout [19].

In line with the job demands–resources theory on the causes of burnout [1,7], the BAT-12 shows a positive relationship with job stressors and a negative relationship with job resources [19,20].

To measure the mediating variables job demands and job resources, we adopted the Workplace Stressors Assessment Questionnaire [21]. This questionnaire is a relatively short and psychometrically sound measure that systematically monitors employees’ perceptions of workplace-related stressors. It contains questions on (i) job demands (e.g., the number of meetings, workload, conflicting demands, neglected tasks and unrealistic time pressure) that positively correlate with overall work stress and (ii) job resources (i.e., control, support, role, relationships and rewards) that negatively correlate with overall work stress [21].

The internal consistency of (the factors) of the Burnout Assessment Tool and the Workplace Stressors Assessment Questionnaire was computed via the Cronbach’s α, which can range from 0.0 to 1.0, and quantifies the degree to which items on an instrument are correlated with one another [22].

### 2.3. Independent Variable and Control Variables

Our independent variable work regime was operationalised with the question “What is your employment percentage? (e.g., 100 = full-time; 50 = half-time)” accompanied with a slider from 0 to 100 allowing the entry of an exact number. We chose to operationalise our independent variable in this way for two reasons, First, we followed previous recommendations to rely on continuous measures instead of arbitrary categories of working time [17]. Second, it allows us to clearly distinguish between “official” work regimes and “unofficial” hours of overtime employees perform. Separately controlling for hours of overtime is crucial because previous studies conclude that chronic overwork is a powerful trigger of burnout [8,23].

Accordingly, the first control variable we included was the number of weekly overtime hours. We operationalised this control variable with the question “How many overtime hours do you perform on average per week?”. Furthermore, the aforementioned study on work regimes and burnout among physicians links being female and younger to an increased risk of burnout [15], whereas the flexibility to choose working hours may reduce the risk of burnout [8,15]. Therefore, we additionally included gender, age and flexibility in working hours as control variables.

### 2.4. Methods for Data Analysis

To test our first hypothesis, we performed a linear regression of work regime on total burnout risk (Equation (1)) estimating the total association (path c) of our conceptual model. Consequently, we introduced the control variables (Equation (2)) and mediators (Equation (3)) to estimate the residual direct association (path c’).

In the following summary of the baseline specification for the first hypothesis *BAT* reflects the dependent variable overall burnout risk, *W* denotes the independent variable work regime. Control variables are presented as follows: *O* = overtime, *G* = gender, *A* = age, *F* = flexibility in working hours. *JD* indicates the mediator job demands and *JR* the mediator job resources. Finally, $\beta_{1}$ is the intercept and ε denotes the error terms. The coefficient estimates are $\beta_{2}$ for the independent variable, $\alpha_{1}$, $\alpha_{2}$, $\alpha_{3}$, $\alpha_{4}$ for the control variables and $\theta_{1}$, $\theta_{2}$ for the mediators. A brief summary of the operationalisation of all variables will also be reported along with the results..

(1) $$BAT_{i}=\beta_{1}+\beta_{2}W_{i}+\varepsilon_{i}$$

(2) $$BAT_{i}=\beta_{1}+\beta_{2}W_{i}+\alpha_{1}O_{i}+\alpha_{2}G_{i}+\alpha_{3}A_{i}+\alpha_{4}F_{i}+\varepsilon_{i}$$

(3) $$BAT_{i}=\beta_{1}+\beta_{2}W_{i}+\alpha_{1}O_{i}+\alpha_{2}G_{i}+\alpha_{3}A_{i}+\alpha_{4}F_{i}+\theta_{1}JD_{i}+\theta_{2}JR_{i}+\varepsilon_{i}$$

To test our second and third hypothesis, we respectively estimated the indirect associations through job demands (path a_1_b_1_) and job resources (path a_2_b_2_) via the ‘Product-of-Coefficients-Method’ for mediation analysis. To obtain the coefficients and standard errors for path a_1_ and path a_2_, we regressed work regime on job demands and job resources while controlling for the same variables as in our baseline specification. The coefficients and standard errors for path b_1_ and b_2_ were already identified in Equation 3.

## 3. Results

### 3.1. Descriptive Analysis

**Figure A1:**
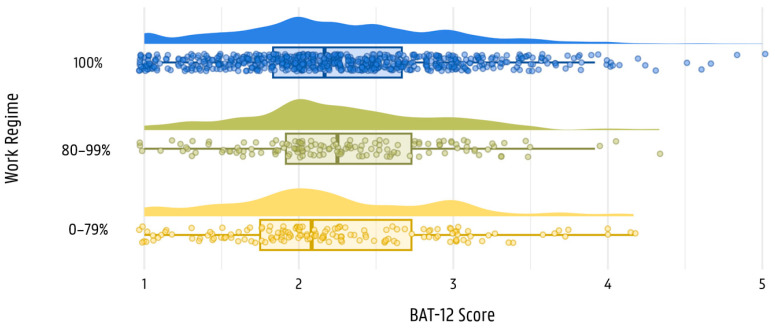
Raincloud plot for work regime and overall burnout risk according to work regime.

As an initial exploration of our dataset, we displayed the descriptive statistics and correlations between part-time work regimes and (i) total BAT-12 score, (ii) factor scores for burnout symptoms exhaustion, mental distance, emotional impairment and cognitive impairment and (iii) WSAQ scores for job demands and job resources in Table 2. A visual representation of the distribution of BAT-12 scores according to types of work regimes can be found in Appendix A (Figure A1).

The average total BAT-12 score among full-time employees (*n* = 714) was 2.266 (SD = 0.718), whereas part-time employees (*n* = 292) scored on average 2.253 (SD = 0.686) for overall burnout risk. As displayed in column “Correlation PT (r_pb_)” in Table 2, we find no correlations between working part-time and total burnout risk (r_pb_ = −0.008) or burnout symptoms exhaustion (r_pb_ = −0.001), mental distance (r_pb_ = −0.005), cognitive impairment (r_pb_ = −0.045) and emotional impairment (r_pb_ = 0.021). Additionally, no correlation between part-time work and job resources was identified (r_pb_ = −0.024). We only observe a very small correlation [22] (r_pb_ = −0.095) between part-time work regimes and job demands.

### 3.2. Regression Analysis

The regression results in Table 3 show no significant total association (path c) between burnout and work regime (Equation (1)). This result remains stable after introducing the control variables (Equation (2)). Similarly, no direct association (path c’) between burnout and work regime and burnout is observed after introducing the mediators (Equation (3)). Therefore, we reject our first hypothesis that there is a significant association between work regimes and burnout in general.

Equation (2) also shows that the control variables overtime, gender and age are significantly associated with burnout as expected (*p* < 0.001). However, we find no significant association between schedule flexibility and burnout (*p* = 0.148).

Furthermore, Equation (3) clarifies that the association between overtime and burnout found in Equation (2) is mediated by job demands. Equation (3) also confirms the theoretical expectation that the mediators job demands (path b_1_) and job resources (path b_2_) are both significantly associated with burnout (*p* = 0.000).

### 3.3. Mediation analysis

**Figure 2:**
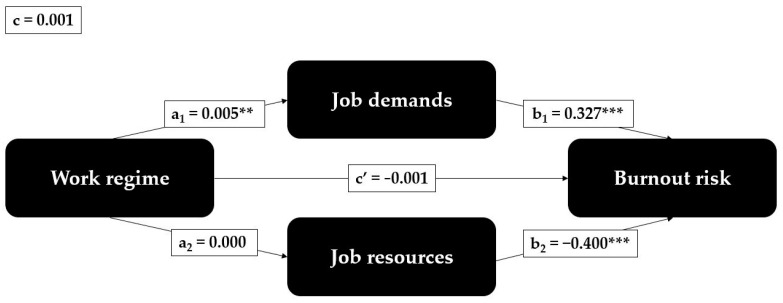
Estimation of the conceptual model. Notes. Presented statistics are the path coefficient estimates. Stars indicate the significance level: *** *p* < 0.001; ** *p* < 0.01.

The product of the path coefficients a_1_ and b_1_ in our estimated model (Figure 2) reveals a significant indirect association between work regime and burnout risk through job demands (path a_1_b_1_, *p* = 0.003). Thus, we accept the second hypothesis that there is a significant indirect association between work regime and burnout through job demands.

However, the product of coefficients a_2_ and b_2_ yields no significant indirect association via job resources (path a_2_b_2_, *p* = 0.720). Therefore, we reject the third hypothesis that there is a significant indirect association between work regime and burnout through job resources.

## 4. Discussion

This study sought to explore associations between work regimes and burnout risk among a heterogenous sample of employees, representative for age and gender. To this end, 1006 participants varying in work regime and job domain completed the Burnout Assessment Tool [5]. We discuss the three major findings of our study below.

First, the results of our descriptive analysis confirmed that part-time work is not accompanied by lower burnout risk and that part-time employees experience the same (degree of) burnout symptoms as their full-time counterparts. These findings are similar to those of previous studies among physicians [15] and teachers [16] that found no objective differences in burnout risk among part-time and full-time employees.

Second, using continuous measures for work regimes and controlling for other variables influencing burnout, the results of our regression analyses confirmed the robustness of our previous findings. In short, they suggest that shorter work regimes are not accompanied by a lower burnout risk. In line with the previous study among physicians [15], our study also confirmed that female and younger individuals were more prone to developing burnout. However, in contrast to the previous study among physicians [15], our participants with schedule flexibility did not experience a lower burnout risk compared to participants with no schedule flexibility.

Third, the results of our mediation analysis only partly confirmed the theoretical expectation that job demands and resources mediate the relationship between work regimes and burnout. More specifically, our mediation results provided no evidence that employees’ job resources differ according to their work regime. However, the mediation results suggest that part-time employees do experience slightly fewer job demands compared to their full-time counterparts.

Since there is no total or direct association between work regimes and burnout (cf. supra) but there is an indirect association via job demands, our findings may raise even more questions about how work regimes and burnout are related.

In this context, the recent extension of the seminal job demands–resources theory [25] points out the major limitation of our study and a clear signpost for future research. More specifically, the recent COVID-19 crisis has shown that job characteristics alone are insufficient to explain employee health [25]. Following the latest recommendations of Demerouti and Bakker, the interplay between demands of the individual, the family, the job and the organisation could be taken into account [25] when studying the relationship between work regimes and burnout.

Since there is an overwhelming amount of evidence that the part-time work is a common strategy for handling family responsibilities [26], it would be interesting to further investigate whether part-time employees’ high family demands balance out their lower job demands—leading to a similar burnout risk as their full-time colleagues.

## 5. Conclusions

In conclusion, although part-time employees experience slightly fewer job demands than their full-time colleagues, shorter work regimes are not accompanied by lower burnout risk. In fact, part-time employees are equally prone to developing job burnout as their full-time colleagues. This indicates that the findings of the previous studies among physicians [15] and teachers [16] can be generalised towards more heterogenous working populations in terms of job domains. These findings tie well with the concerns that working time reduction may not per se reduce the risk of burnout [13,14]. However, two additional limitations of our study are its explorative character and its cross-sectional survey design. Therefore, a thorough (quasi-)experimental evaluation of burnout prevention focused on mere work regimes (e.g., offering part-time options and shorter weeks) is needed. Since positive intervention effects generally diminish with time in the context of burnout prevention [27], we strongly recommend implementing longitudinal designs with multiple measurement moments. In that way, future burnout prevention intervention studies can investigate the long-term effects of offering part-time options and shorter working weeks on employees’ overall burnout risk.

Meanwhile, although not the main focus of our study, our mediation results underwrite that job demands and resources still play a crucial role in the development of burnout. Even more so, our regression results suggest that the relationship between overtime and burnout is explained by the high job demands that overworking employees experience. These findings naturally raise the question whether merely offering working time reductions without changing the job demands makes sense in the context of burnout prevention. So instead of focusing en masse on working time “as such”, it might be more useful to address the high job demands causing chronic overtime and burnout in both part-time and full-time workers.

## Figures and Tables

**Table 1:** Sample specification.

Participants’ Characteristics	Absolute Frequency (Number)	Relative Frequency (in %)
***Gender***		
Male	493	49.01%
Female	513	50.99%
***Work regime***		
Full-time	714	70.97%
Part-time	292	29.03%
***Sector***		
Private sector	709	70.48%
Public sector	297	29.52%
***Organisation size***		
Small/medium organisation	503	50.00%
Large organisation	503	50.00%
***Job domain***		
Administration	137	13.62%
Agriculture	4	0.40%
Communication	13	1.29%
Construction	30	2.98%
Creative	4	0.40%
Education	105	10.44%
Finance	52	5.17%
Government Services	80	7.95%
Health	106	10.54%
Hospitality and tourism	24	2.39%
Human resources	20	1.99%
IT	66	6.56%
Legal	11	1.09%
Logistics and transport	62	6.16%
Maintenance	15	1.49%
Management	13	1.29%
Marketing	4	0.40%
Production	65	6.46%
Purchase	9	0.89%
Research and development	26	2.58%
Sales	52	5.17%
Service	83	8.25%
Technics	25	2.49%

**Table 2:** Descriptive analysis of the independent variable (burnout risk) and mediating variables (job demands and resources).

Scales Measuring Outcomes	Cronbach’s α	Part-Time Employees	Full-Time Employees	Correlation Part-Time (r_pb_)
Total BAT-12	0.93	M = 2.253 (SD = 0.686)	M = 2.266 (SD = 0.718)	−0.008
BAT-12 Exhaustion	0.87	M = 2.587 (SD = 0.854)	M = 2.590 (SD = 0.922)	−0.001
BAT-12 Mental distance	0.86	M = 2.228 (SD = 0.863)	M = 2.238 (SD = 0.898)	−0.005
BAT-12 Cognitive impairment	0.86	M = 2.209 (SD = 0.737)	M = 2.284 (SD = 0.777)	−0.045
BAT-12 Emotional impairment	0.89	M = 1.990 (SD = 0.807)	M = 1.951 (SD = 0.812)	0.021
Total WSAQ Demands	0.86	M = 2.338 (SD = 0.913)	M = 2.537 (SD = 0.957)	−0.095 *
Total WSAQ Resources	0.91	M = 3.449 (SD = 0.653)	M = 3.481 (SD = 0.601)	−0.024

Notes. Presented statistics in column “Cronbach’s α” are Cronbach’s alphas that measure the internal consistency of the respective (sub)scales: 12-item version of the Burnout Assessment Tool (BAT-12) and the Workplace Stressors Assessment Questionnaire (WSAQ). In the columns “Part-time” and “Full-time”, presented statistics are means (M) with their standard deviations (SD) in between parentheses. Presented statistics in column r_pb_ are point biserial correlation coefficients for working part-time (0 = no, 1 = yes). The star (*) indicates an effect size of r_pb_ ≥ 0.05 [24].

**Table 3:** Regression analysis of the dependent variable burnout risk on the independent variable work regime.

Burnout Risk (Total BAT-12 Score)	Equation (1)	Equation (2)	Equation (3)
Work regime (% of full-time)	0.001 (0.001)	0.001 (0.001)	−0.001 (0.001)
Overtime (hours per week)		0.012 *** (0.004)	−0.001 (0.003)
Gender (0 = male, 1 = female)		0.178 *** (0.047)	0.105 ** (0.035)
Age (in years)		−0.009 *** (0.002)	−0.007 *** (0.001)
Schedule flexibility (0 = no, 1 = yes)		−0.066 (0.046)	0.053 (0.035)
Job demands (mean WSAQ score demands)			0.327 *** (0.020)
Job resources (mean WSAQ score resources)			−0.400 *** (0.030)

Notes: The columns represent the three equations of our baseline specifications as defined in Section 2.4. Presented statistics are coefficient estimates with standard errors between parentheses. Stars indicate the significance level: *** *p* < 0.001; ** *p* < 0.01.

## Data Availability

The data and STATA-15 code from this study are available on request from the corresponding author.
